# Mechanical Stretch Triggers Epithelial-Mesenchymal Transition in Keratinocytes Through Piezo1 Channel

**DOI:** 10.3389/fphys.2022.745572

**Published:** 2022-05-09

**Authors:** Jiahao He, Shengzhou Shan, Qingfeng Li, Bin Fang, Yun Xie

**Affiliations:** Department of Plastic and Reconstructive Surgery, Shanghai Ninth People’s Hospital, Shanghai Jiao Tong University School of Medicine, Shanghai, China

**Keywords:** Piezo1, epithelial-mesenchymal transition, mechanical force, keratinocyte, epithelial physiology

## Abstract

The epithelial-mesenchymal transition (EMT) process has emerged as a central regulator of embryonic development, tissue repair and tumor malignancy. In recent years, researchers have specifically focused on how mechanical signals drive the EMT program in epithelial cells. However, how epithelial cells specifically leverage mechanical force to control the EMT process remains unclear. Here, we show that the *bona fide* mechanically activated cation channel Piezo1 plays a critical role in the EMT. The Piezo1 is expressed in human primary epidermal keratinocytes (HEKs) and is responsible for the mechanical stretch-induced Ca^2+^ concentration. Inhibition of Piezo1 activation by the inhibitor GsMTx4 or by siRNA-mediated Piezo1 knockdown influenced the morphology and migration of HEKs. Moreover, Piezo1 activity also altered EMT-correlated markers expression in response to mechanical stretch. We propose that the mechanically activated cation channel Piezo1 is an important determinant of mechanical force-induced EMT in keratinocytes and might play similar roles in other epithelial cells.

## Introduction

The transition of epithelial cells into mesenchymal cells, which is a cellular mechanism referred to as epithelial-mesenchymal transition (EMT), plays a crucial role in tissue repair, organ fibrosis and cancer progression (Thiery et al., 2009). In EMT, epithelial cells lose their polarity and cell-cell adhesion, regulate the expression of various EMT biomarkers and acquire mesenchymal phenotypes, such as migration and invasion (Lamouille et al., 2014). EMT can be activated by several differentiation factors, including transforming growth factor-β (TGFβ) (Xu et al., 2009), WNTs (Savagner, 2001) and mitogenic growth factors (Uttamsingh et al., 2008). In recent years, there has been increasing evidence that mechanical force also serves as a key regulator of EMT (Zhou et al., 2015; Przybyla et al., 2016; Zhou et al., 2020). Some mechanosensitive molecules, such as integrins (Yilmaz and Christofori, 2009), cadherin complexes (Sim et al., 2015) and ion channels (Azimi and Monteith, 2016), are capable of sensing and integrating mechanical force to induce EMT. However, our knowledge of the mechanical control of EMT is still unclear, and the molecular mechanisms linking mechanical force with EMT remain rudimentary.

The recent discovery of a novel mechanically activated cation channel, Piezo1 (Coste et al., 2010), led us to consider whether Piezo1 mediated EMT in response to mechanical force. Piezo1 is expressed in a diverse set of cells and tissues within mammals, modulating a multitude of physiological functions, including innate immunity (Solis et al., 2019), gut disorders (Sugisawa et al., 2020) and aging (Segel et al., 2019). Notably, previous studies have emphasized the important role of Piezo1 in regulating the physiological functions of epithelial cells (Stewart and Davis, 2019). For example, activation of Piezo1 could trigger a fast proliferative response in epithelial cells, thereby acting as a mechanosensor to control epithelial homeostasis (Gudipaty et al., 2017). Mechanical stretch also stimulated ATP release from alveolar type I (ATI) cells *via* Piezo1 (Diem et al., 2020). Furthermore, the activity of Piezo1 promoted MCF-7 cells (a human breast epithelial cell line) migration and invasion, underscoring a potent role of Piezo1 in breast cancer progression (Li et al., 2015). Although there has been progress in the research on Piezo1-mediated epithelial cell behaviors, the involvement of Piezo1 in EMT has not been investigated to date.

In this study, we found that human primary epidermal keratinocytes (HEKs) expressed the Piezo1 and that Piezo1 activation mediated calcium (Ca^2+^) influx in response to mechanical stretch. In the context of mechanical stretch, inhibition or knockdown of the Piezo1 not only changed the morphology and migration of HEKs but also altered the expression of EMT-associated markers.

## Materials and Methods

### Cell Culture and Treatment

The human primary epidermal keratinocytes (HEKs) were purchased from ScienCell Research Laboratories. The HEKs were cultured with keratinocyte medium (ScienCell Research Laboratories, Carlsbad, CA, United States) at 37°C with 5% CO_2_. The medium was changed every 3 days. We used HEKs from passages three to five.

### Application of Mechanical Stretch

**FIGURE 1:**
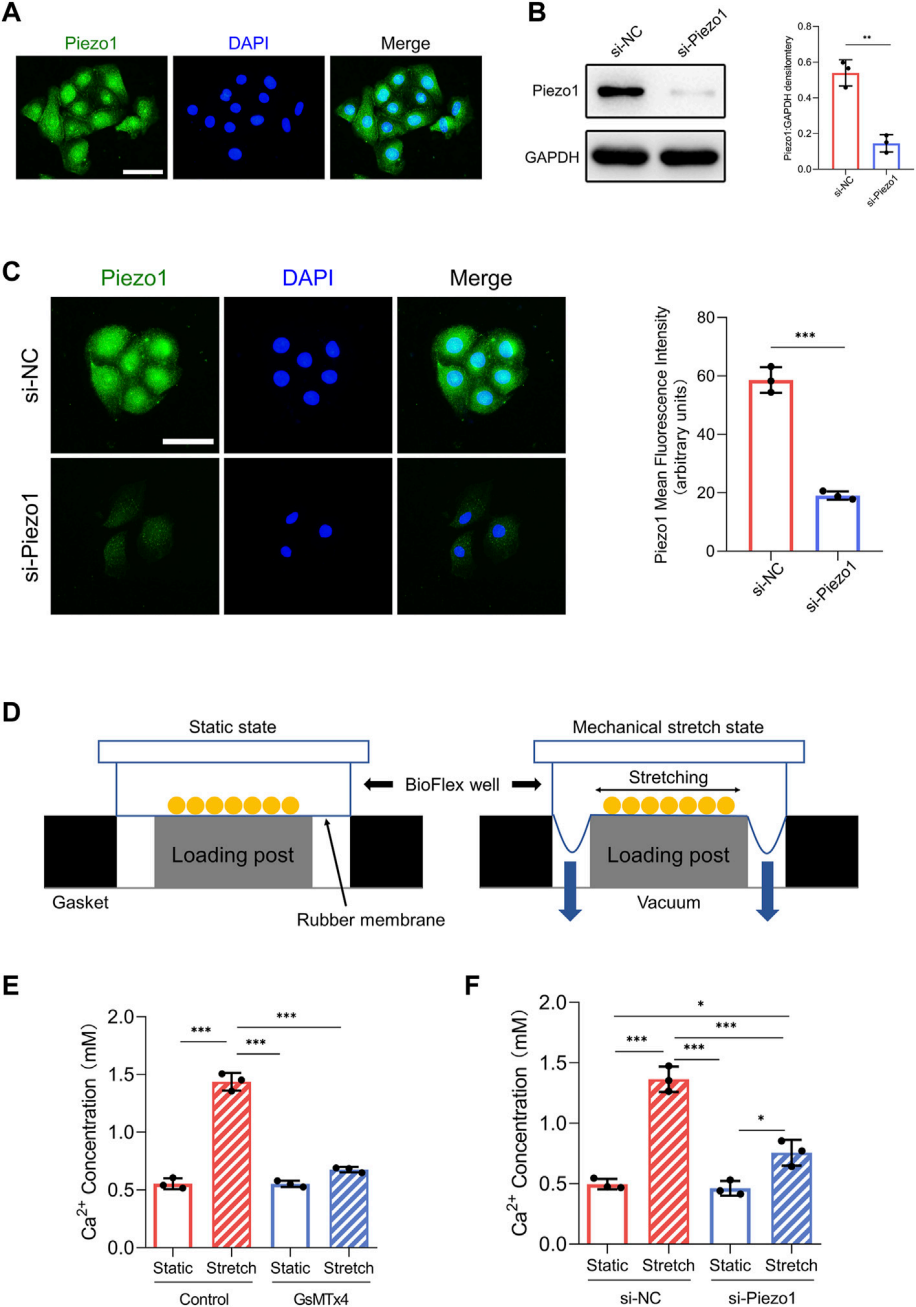
Piezo1 expression and function in HEKs. **(A)** The expression of Piezo1 in HEKs was analyzed by immunofluorescence. (Scale bar = 50 μm). **(B)** After siPiezo1 transfection, protein expression level of Piezo1 was decreased analyzing by Western blotting. **(C)** After siPiezo1 transfection, protein expression level of Piezo1 was decreased analyzing by immunofluorescence. **(D)** Schematic of Flexcell Tension system. **(E)** Ca^2+^ concentration in HEKs was determined by calcium colorimetric detection kit in GsMTx4‐treated condition. GsMTx4 treatment inhibited the Piezo1-induced calcium influx. **(F)** Ca^2+^ concentration in HEKs was determined by calcium colorimetric detection kit in Piezo1 siRNA‐treated condition. Piezo1-siRNA treatment inhibited the Piezo1-induced calcium influx. The results are expressed as the means with SD (*n* = 3). *p < 0.05, **p < 0.01, ***p < 0.005.

HEKs were seeded on six-well flexible silicone rubber BioFlex plates (Flexcell International, Burlington, NC, United States) at a density of 5 × 10^5^ cells/well in 2 ml of medium. Cells were cultured for 24 h to reach 60–80% confluence before mechanical stretch was applied. Cyclic mechanical stretch was applied with 10% amplitude at 0.5 Hz for 24 h by using an FX-5000T Flexcell Tension Plus device (Flexcell International, Burlington, NC, United States) as previously reported (Zhou et al., 2015). HEKs cultured in the same plates but left non‐stretched served as controls. The diagram of cyclic mechanical stretch device is shown in Figure 1D.

### Western Blotting

Total proteins were extracted from cells by using Radio immune precipitation assay (RIPA) lysis buffer. Concentrations of protein were detected by the bicinchoninic acid (BCA) assay (Thermo Fisher Scientific). 10 μg of total protein were separated by 10% SDS-PAGE, followed by transfer to PVDF membranes (Millipore, United States). The membranes were blocked with 5% bovine serum albumin at room temperature for 1 h and then probed with primary antibodies against Piezo1 (1:1000; SAB), fibronectin, Vimentin (1:1000, all from Abcam, Cambridge, United Kingdom), MMP9, E-cadherin, N-cadherin (1:5000, all from Abcam), MMP2, α-SMA, GAPDH (1:1000, all from Cell Signaling Technology, Danvers, MA, United States). Next day, after washing with TBST 10 min for three times, bands were then incubated with secondary antibodies and visualized using an ECL detection system (Millipore, Bedford, MA, United States). ImageJ software (National Institutes of Health, Bethesda, MD, United States) was used for quantitative analysis of immunoreactive bands.

### siRNA and Transfection

For Piezo1 silencing, HEKs were transfected in six-well plates with 100 nM Piezo1 siRNA by using Lipofectamine RNAiMAX reagent (Invitrogen, Carlsbad, CA, United States) according to the manufacturer’s protocol. The sequences were as follows: Piezo1-siRNA, 5′-AGA​AGA​AGA​UCG​UCA​AGU​ATT-3′ (sense) and 5′-UAC​UUG​ACG​AUC​UUC​UUC​UTT-3′ (antisense), negative control (NC) siRNA, 5′-GUG​AGC​GUC​UAU​AUA​CCA​UTT-3′ (sense) and 5′-AUG​GUA​UAU​AGA​CGC​UCA​CTT-3′ (antisense). The sequences used were self-selected.

### Piezo1 Inhibitor Treatment

The Piezo1 inhibitor GsMTx4 (Alomone Labs, Jerusalem, Israel) was purchased and dissolved in PBS solution. 5 µM GsMTx4 was used for all experiments according to the manufacturer’s protocol. The incubation time with GsMTx4 is 24 h, accompanying by stretch process.

### Calcium Assay

To analyze calcium concentration, the calcium assay kit (Abcam, Cambridge, MA, United States) was purchased. Before calcium detection, cell numbers in every group exhibit no statistical difference. The cell lysates collected were used for analyzing cytosolic calcium level. Measurement was performed in a 96-well plate, at 575 nm by using an Infinite M200 Pro microplate reader (Tecan, Männedorf, Switzerland).

### Assays for Cell Migration

Migration assays were performed using Transwell chambers (Corning, Tewksbury, MA, United States) as described previously (Fang et al., 2019). HEKs were seeded in keratinocyte medium without keratinocyte growth supplement (KGS) in the upper chambers. The lower chambers were filled with keratinocyte medium. After 24 h, the migrated HEKs were fixed and stained for 20 min in a 0.1% crystal violet solution. Images of migrated HEKs on the lower filters within three random fields were captured with a microscope. Migrated HEKs numbers were calculated by the ImageJ software.

### Immunofluorescence

Cell samples were fixed in 4% paraformaldehyde for 20 min at room temperature. Cell samples were then washed, permeabilized and blocked. Antibodies used for immunofluorescence staining were anti-Piezo1 (1:100; SAB), anti-F-actin (1:200, Abcam, Cambridge, United Kingdom), anti-N-cadherin (1:200, Abcam), anti-vimentin (1:200, Abcam), anti-αSMA (1:200, Cell Signaling Technology) an Alexa Fluor 488-conjugated goat anti-mouse secondary antibody (1:200; Jackson ImmunoResearch) and an Alexa Fluor 594-conjugated goat anti-rabbit secondary antibody (1:200; Jackson ImmunoResearch). For phalloidin staining, cells were incubated with Alexa Fluor 647 Phalloidin (Cytoskeleton, Inc., Denver, CO, United States, 1:200) for 30 min at room temperature. Subsequently, samples were stained with DAPI (Solarbio, Beijing, China). Images were captured using a Nikon Eclipse E800 microscope (Nikon, Melville, NY, United States) and a Zeiss 710 laserscanning microscope (Zeiss, Thornwood, NY, United States).

### Statistical Analysis

Data are presented as the mean ± SD. Statistical differences among groups were assessed using a two-tailed Student’s *t*-test or one-way ANOVA. *p* < 0.05 was considered statistically significant.

## Results

### HEKs Sense Mechanical Stretch *Via* Piezo1

First, we investigated whether HEKs express the Piezo1 protein. Immunofluorescence analysis illustrated the presence of the Piezo1 protein in HEKs (Figure 1A). Furthermore, because Piezo1 is a transmembrane cation channel that facilitates Ca^2+^ influx in response to mechanical force (Coste et al., 2010), we tested whether Piezo1 affected Ca^2+^ entry in HEKs by applying the Piezo1 inhibitor GsMTx4 (Bae et al., 2011) and siRNA-mediated Piezo1 knockdown. Western blot and immunofluorescence analyses confirmed the efficiency of siRNA transfection in HEKs (Figures 1B,C). Importantly, in HEKs, the increase in Ca^2+^ concentration induced by mechanical stretch was inhibited by GsMTx4 treatment or Piezo1 knockdown (Figures 1E,F). Collectively, these data indicate that HEKs express Piezo1 and sense mechanical stretch through Piezo1.

### Mechanical Stretch Influences the Morphology of HEKs Through Piezo1

**FIGURE 2:**
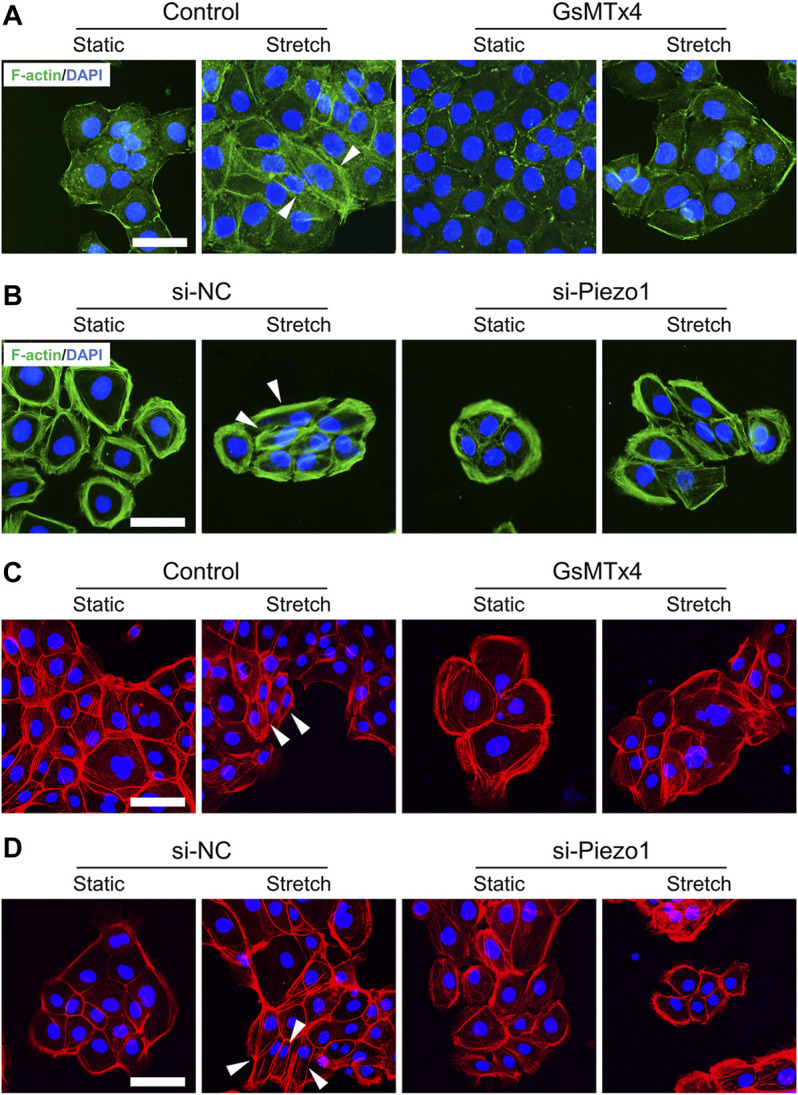
Mechanical stretch influences HEKs morphology through Piezo1. **(A)** Representative images of HEKs in GsMTx4‐treated condition by F-actin staining. GsMTx4 treatment inhibited the Piezo1-induced cellular mesenchymal cell morphology. **(B)** Representative images of HEKs in Piezo1 siRNA‐treated condition by F-actin staining. Piezo1-siRNA treatment inhibited the Piezo1-induced cellular mesenchymal cell morphology. **(C)** Representative images of HEKs in GsMTx4‐treated condition by phalloidin staining. GsMTx4 treatment inhibited the Piezo1-induced cellular mesenchymal cell morphology. **(D)** Representative images of HEKs in Piezo1 siRNA‐treated condition by phalloidin staining. Piezo1-siRNA treatment inhibited the Piezo1-induced cellular mesenchymal cell morphology. (Scale bar: 50 μm).

To analyze whether Piezo1 is involved in changes in EMT phenotypes, we first tested the effect of Piezo1 activity on cell morphology in HEKs. As expected, some stretched HEKs showed spindle shapes. Meanwhile, cells treated with GsMTx4 or Piezo1 knockdown exhibited polygonal shapes similar to static cells when subjected to mechanical stretch (Figures 2A–D). These findings demonstrate the significant role of Piezo1 in modulating the morphology of HEKs.

### Mechanical Stretch Improved the Migration of HEKs Through Piezo1

**FIGURE 3:**
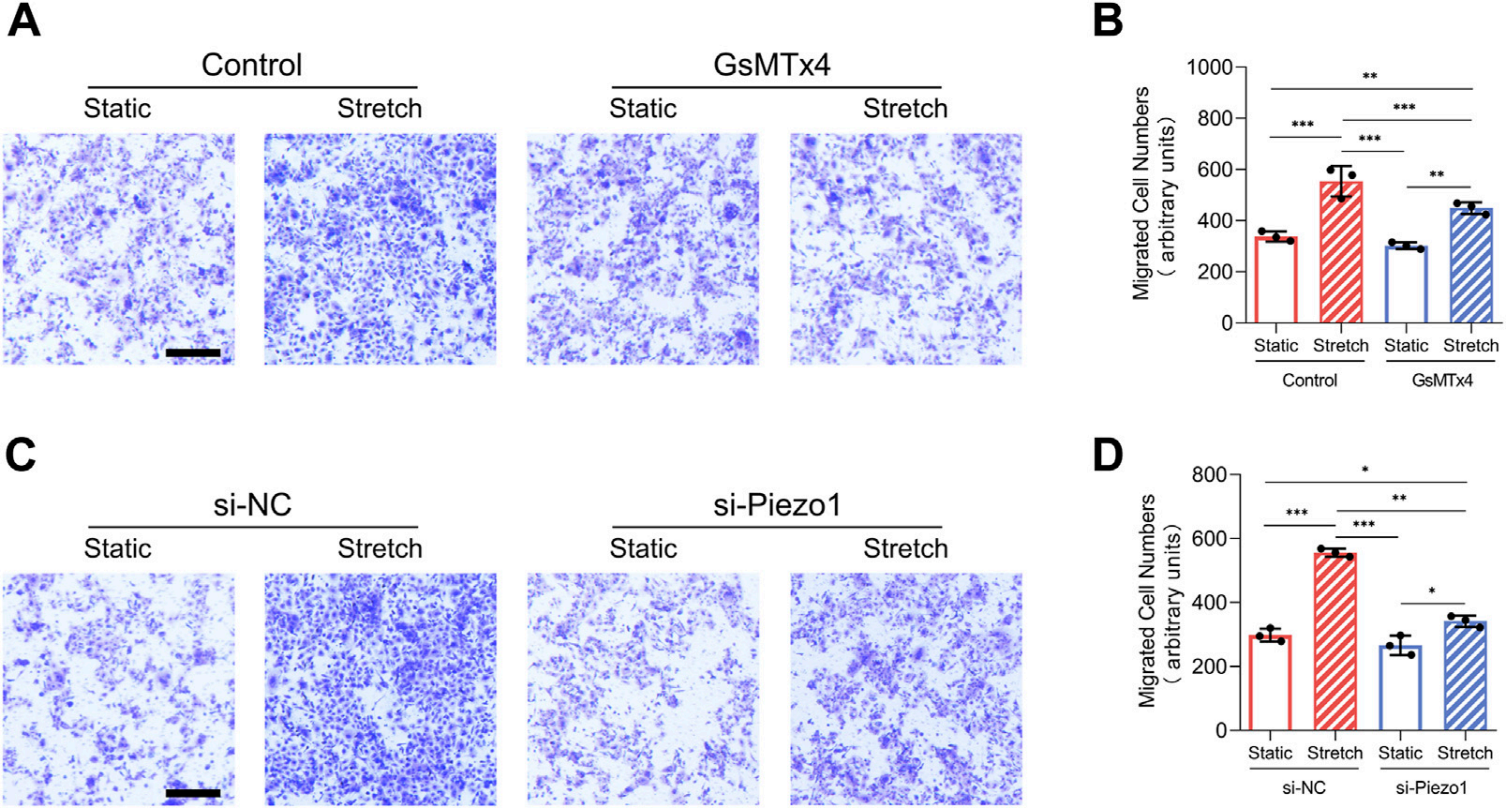
Mechanical stretch alters HEKs migration by activating Piezo1. **(A)** Representative images of migrated HEKs in GsMTx4‐treated condition. GsMTx4 treatment inhibited the Piezo1-induced cell migration. (Scale bar: 100 μm). **(B)** Quantitative analysis of the number of migrated cells. **(C)** Representative images of migrated HEKs in Piezo1 siRNA‐treated condition. Piezo1-siRNA treatment inhibited the Piezo1-induced cell migration. (Scale bar: 100 μm). **(D)** Quantitative analysis of the number of migrated cells. The results are expressed as the means with SD (*n* = 3). *p < 0.05, **p < 0.01, ***p < 0.005.

Increased migration has been heralded as a key event of EMT (Mittal, 2018). In our research, mechanical stretch-induced migration of HEKs was decreased by GsMTx4 application or Piezo1 knockdown (Figures 3A–D). These results indicate that mechanical stretch-induced HEKs migration might be regulated by Piezo1 activity.

### Mechanical Stretch Regulates EMT Markers Expression in HEKs *Via* Piezo1

**FIGURE 4:**
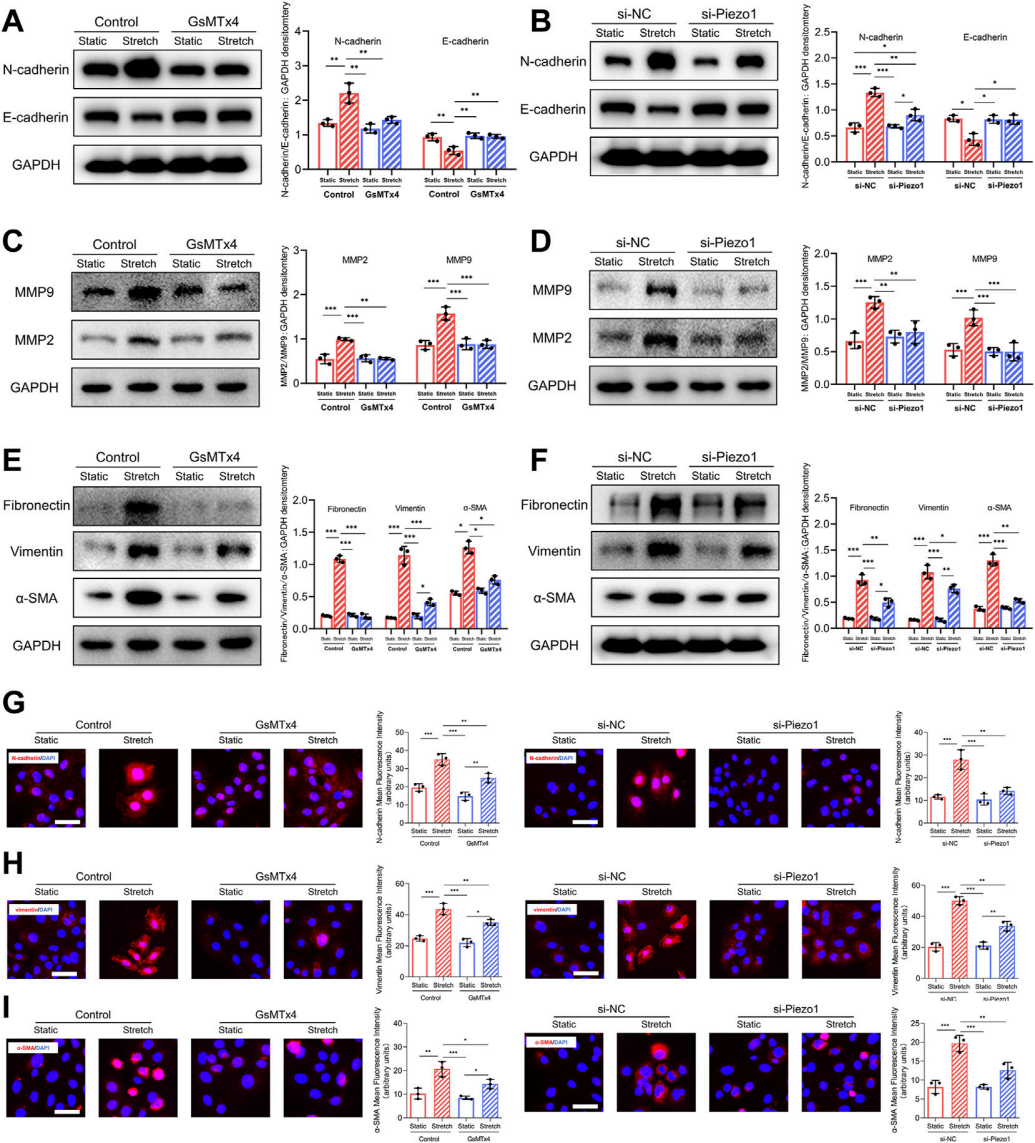
Mechanical stretch changes EMT-associated biomarkers expression through Piezo1. **(A)** Western blotting analysis of E-cadherin and N-cadherin in HEKs in GsMTx4‐treated condition. GsMTx4 treatment inhibited the Piezo1-induced increased expression of N-cadherin and decreased expression of E-cadherin. **(B)** Western blotting analysis of E-cadherin and N-cadherin in HEKs in Piezo1 siRNA‐treated condition. Piezo1-siRNA treatment inhibited the Piezo1-induced increased expression of N-cadherin and decreased expression of E-cadherin. **(C)** Western blotting analysis of MMP2 and MMP9 in HEKs in GsMTx4‐treated condition. GsMTx4 treatment inhibited the Piezo1-induced increased expression of MMP2 and MMP9. **(D)** Western blotting analysis of MMP2 and MMP9 in HEKs in Piezo1 siRNA‐treated condition. Piezo1-siRNA treatment inhibited the Piezo1-induced increased expression of MMP2 and MMP9. **(E)** Western blotting analysis of α-SMA, vimentin and fibronectin in HEKs in GsMTx4‐treated condition. GsMTx4 treatment inhibited the Piezo1-induced increased expression of α-SMA, vimentin and fibronectin. **(F)** Western blotting analysis of α-SMA, vimentin and fibronectin in HEKs in Piezo1 siRNA‐treated condition. Piezo1-siRNA treatment inhibited the Piezo1-induced increased expression of α-SMA, vimentin and fibronectin. **(G)** Representative images of N-cadherin in GsMTx4‐treated and Piezo1 siRNA‐treated HEKs. GsMTx4 and Piezo1 siRNA treatment inhibited the Piezo1-induced increased expression of N-cadherin. **(H)** Representative images of vimentin in GsMTx4‐treated and Piezo1 siRNA‐treated HEKs. GsMTx4 and Piezo1 siRNA treatment inhibited the Piezo1-induced increased expression of vimentin. **(I)** Representative images of α-SMA in GsMTx4‐treated and Piezo1 siRNA‐treated HEKs. GsMTx4 and Piezo1 siRNA treatment inhibited the Piezo1-induced increased expression of α-SMA (Scale bar: 50 μm). The results are expressed as the means with SD (*n* = 3). *p < 0.05, **p < 0.01, ***p < 0.005.

It has been reported that a series of biomarkers are associated with the EMT process (Zeisberg and Neilson, 2009). In our study, we observed that the expression of E-cadherin, a marker of cell-cell adhesion, was downregulated after mechanical stretch, whereas the expression of N-cadherin was upregulated. Significantly, the changes in E-cadherin and N-cadherin expression in response to mechanical stretch were inhibited after blockade and knockdown of Piezo1 (Figures 4A,B). Matrix metalloproteinase 2 (MMP2) and MMP9 are hallmarks of EMT and promote cell migration (Nisticò et al., 2012). Our data showed that the mechanical stretch-induced upregulation of MMP2 and MMP9 was decreased by GsMTx4 and Piezo1 knockdown (Figures 4C,D). Increased expression of α‐smooth muscle actin (α‐SMA) and vimentin are also mesenchymal features that develop during the EMT process (Huang et al., 2012). In our study, mechanical stretch-induced α‐SMA and vimentin upregulation was alleviated in HEKs by the inhibition or knockdown of Piezo1 (Figures 4E,F). Another hallmark of EMT is the upregulation of extracellular matrix (ECM) proteins to reinforce ECM remodeling (Gonzalez and Medici, 2014). Piezo1 inhibition or knockdown inhibited the mechanical stretch-induced increase in fibronectin (Figures 4E,F). Additionally, immunofluorescence staining was conducted to further substantiate the changes in N-cadherin, vimentin and α‐SMA, and the result was consistent with that obtained by western blotting (Figures 4G–I). Therefore, we concluded from these results that Piezo1 was involved in mechanical stretch-induced changes in EMT biomarkers.

## Discussion

Our findings demonstrate for the first time that Piezo1 serves as a key regulator of mechanical force-induced EMT in HEKs. First, this study showed that HEKs expressed Piezo1 and sensed mechanical stretch through Piezo1. Furthermore, the activation of Piezo1 influenced HEKs shapes and migration in response to mechanical stretch. Finally, mechanical stretch regulated EMT markers expression in HEKs through Piezo1. Overall, these findings demonstrated the significant role of Piezo1 in regulating mechanical stretch-mediated EMT processes in HEKs.

Previous reports show that Piezo1 is expressed in different epithelial cell types (Eisenhoffer et al., 2012). Notably, increasing attention has focused on the emerging roles of Piezo1 in the physiology and development of mammalian epithelia (Gudipaty and Rosenblatt, 2017; Stewart and Davis, 2019). However, the function of Piezo1 in mechanical force-mediated EMT, has not been investigated. Similarly, we found that Piezo1 was expressed in HEKs. In addition, Piezo1-mediated Ca^2+^ influx has been described in various mechanosensing cells (Wang and Xiao, 2018). In our study, we confirmed that mechanical stretch mediated Ca^2+^ influx in HEKs through Piezo1. Ca^2+^ signaling could serve as a major second messenger to modulate epithelial cell function and survival (Hoenderop et al., 2005). Importantly, it has been reported that Ca^2+^ is critical for regulating the EMT process in mouse epidermal keratinocytes (Sharma et al., 2019). Taken together, our findings suggest that mechanical stretch promotes Ca^2+^ influx in HEKs through Piezo1, which might lead to EMT.

The alteration of cellular morphology and the acquisition of migration are the key events in EMT (Qin et al., 2005). The effect of Piezo1 on cellular morphology or migration has been reported in several cell types. Piezo1 could modulate cellular cytoskeleton through activation of integrins pathways, Ca^2+^ pathways and calpain 2 (Nourse and Pathak, 2017). Activation of Piezo1 in transformed mouse fibroblasts 3T3B-SV40 (Chubinskiy-Nadezhdin et al., 2019) and optic nerve head astrocytes (Liu et al., 2021) leads to change in cellular morphology by triggering cells redistribution of F-actin cytoskeleton. Activation of Piezo1 also stimulates cellular migration in fibroblasts (He et al., 2021) and mesenchymal stem cells (Mousawi et al., 2020). In our study, mechanical stretch influenced keratinocytes morphology and promoted HEKs migration *via* Piezo1. To facilitate such behaviors, epithelial cells might alter the expression of certain cell junctions proteins (N-cadherin/E-cadherin) (Zhou et al., 2015; Zhou et al., 2020) and matrix metalloproteinases (Orlichenko and Radisky, 2008). Similarly, previous studies have emphasized the role of Piezo1 in regulating VE-cadherin (Friedrich et al., 2019) and MMP2 expression (Kang et al., 2019). Thus, our research confirmed that Piezo1 regulated genes encoding cell junctions and proteases, subsequently contributing to migration in HEKs. The activation of genes encoding cytoskeletal and ECM proteins also contributes to EMT (Lee and Nelson, 2012), such as α-SMA, vimentin and fibronectin. Fibronectin is a glycoprotein that serves as a scaffold for extracellular matrix and has been used as a marker of EMT (Zeisberg and Neilson, 2009). Increased levels of fibronectin have been reported during EMT process in fibrogenesis and cancer progression (Yang et al., 2007). Vimentin and αSMA were cytoskeletal markers of mesenchymal cells (Eckes et al., 2000). Increased expression of vimentin and αSMA largely exhibit the switch from epithelial cell to mesenchymal cells. Our data demonstrated that mechanical stretch stimulates the expression of α-SMA, vimentin and fibronectin by activating Piezo1, which is consistent with previous report that Piezo1 activity stimulates α-SMA and fibronectin in dermal fibroblasts (He et al., 2021). Furthermore, we speculate that the EMT process in keratinocytes largely dependent on calcium signals through Piezo1 activity. Several studies have identified calcium channel as a crucial in modulating EMT process. For example, calcium channel TRPM7 silencing inhibited the EMT in ovarian cancer by attenuating the calcium signals (Liu et al., 2019). This article pointed that calcium signals could regulate E-cadherin and vimentin. The activation of another calcium channel-orai1 also promote EMT process (increased expression of fibronectin and αSMA) in fibrosis (Mai et al., 2016). Calcium could also modulate the expression of MMP2 (Yu-Ju Wu et al., 2020) and MMP9 (Li et al., 2019). Overall, our study indicated that Piezo1 activity induced EMT processes in response to mechanical stretch.

In summary, our research offers the first indication (to our knowledge) that Piezo1 mediates the mechanical control of EMT *in vitro*. However, the role of Piezo1 in the mechanical control of EMT in other cell types, particularly cancer cells, has not been investigated to date. More importantly, future research focused on Piezo1-mediated EMT in embryonic development, fibrosis and cancer progression might increase our knowledge of how mechanical force controls EMT.

## Data Availability

The original contributions presented in the study are included in the article/Supplementary Material, further inquiries can be directed to the corresponding authors.

## References

[B1] Azimi I. Monteith G. R. (2016). Plasma Membrane Ion Channels and Epithelial to Mesenchymal Transition in Cancer Cells. Endocrine-related cancer 23 (11), R517–R525. 10.1530/erc-16-0334 27619258

[B2] Bae C. Sachs F. Gottlieb P. A. (2011). The Mechanosensitive Ion Channel Piezo1 Is Inhibited by the Peptide GsMTx4. Biochemistry 50 (29), 6295–6300. 10.1021/bi200770q 21696149 PMC3169095

[B3] Chubinskiy-Nadezhdin V. I. Vasileva V. Y. Vassilieva I. O. Sudarikova A. V. Morachevskaya E. A. Negulyaev Y. A. (2019). Agonist-induced Piezo1 Activation Suppresses Migration of Transformed Fibroblasts. Biochem. biophysical Res. Commun. 514 (1), 173–179. 10.1016/j.bbrc.2019.04.139 31029419

[B4] Coste B. Mathur J. Schmidt M. Earley T. J. Ranade S. Petrus M. J. (2010). Piezo1 and Piezo2 Are Essential Components of Distinct Mechanically Activated Cation Channels. Science 330 (6000), 55–60. 10.1126/science.1193270 20813920 PMC3062430

[B5] Diem K. Fauler M. Fois G. Hellmann A. Winokurow N. Schumacher S. (2020). Mechanical Stretch Activates Piezo1 in Caveolae of Alveolar Type I Cells to Trigger ATP Release and Paracrine Stimulation of Surfactant Secretion from Alveolar Type II Cells. FASEB J. 34, (9). 12785–12804. 10.1096/fj.202000613rrr 32744386

[B6] Eckes B. Colucci-Guyon E. Smola H. Nodder S. Babinet C. Krieg T. (2000). Impaired Wound Healing in Embryonic and Adult Mice Lacking Vimentin. J. Cell Sci 113 (Pt 13), 2455–2462. 10.1242/jcs.113.13.2455 10852824

[B7] Eisenhoffer G. T. Loftus P. D. Yoshigi M. Otsuna H. Chien C.-B. Morcos P. A. (2012). Crowding Induces Live Cell Extrusion to Maintain Homeostatic Cell Numbers in Epithelia. Nature 484 (7395), 546–549. 10.1038/nature10999 22504183 PMC4593481

[B8] Fang B. Liu Y. Zheng D. Shan S. Wang C. Gao Y. (2019). The Effects of Mechanical Stretch on the Biological Characteristics of Human Adipose‐derived Stem Cells. J. Cell Mol Med 23 (6), 4244–4255. 10.1111/jcmm.14314 31020802 PMC6533502

[B9] Friedrich E. E. Hong Z. Xiong S. Zhong M. Di A. Rehman J. (2019). Endothelial Cell Piezo1 Mediates Pressure-Induced Lung Vascular Hyperpermeability via Disruption of Adherens Junctions. Proc. Natl. Acad. Sci. U.S.A. 116 (26), 12980–12985. 10.1073/pnas.1902165116 31186359 PMC6600969

[B10] Gonzalez D. M. Medici D. (2014). Signaling Mechanisms of the Epithelial-Mesenchymal Transition. Sci. Signal. 7 (344), re8. 10.1126/scisignal.2005189 25249658 PMC4372086

[B11] Gudipaty S. A. Lindblom J. Loftus P. D. Redd M. J. Edes K. Davey C. F. (2017). Mechanical Stretch Triggers Rapid Epithelial Cell Division through Piezo1. Nature 543 (7643), 118–121. 10.1038/nature21407 28199303 PMC5334365

[B12] Gudipaty S. A. Rosenblatt J. (2017). Epithelial Cell Extrusion: Pathways and Pathologies. Semin. Cel. Dev. Biol. 67, 132–140. 10.1016/j.semcdb.2016.05.010 PMC5116298 27212253

[B13] He J. Fang B. Shan S. Xie Y. Wang C. Zhang Y. (2021). Mechanical Stretch Promotes Hypertrophic Scar Formation through Mechanically Activated Cation Channel Piezo1. Cell Death Dis 12 (3), 226. 10.1038/s41419-021-03481-6 33649312 PMC7921104

[B14] Hoenderop J. G. J. Nilius B. Bindels R. J. M. (2005). Calcium Absorption across Epithelia. Physiol. Rev. 85 (1), 373–422. 10.1152/physrev.00003.2004 15618484

[B15] Huang R. Y. Guilford P. Thiery J. P. (2012). Early Events in Cell Adhesion and Polarity during Epithelial-Mesenchymal Transition. J. Cell Sci 125 (Pt 19), 4417–4422. 10.1242/jcs.099697 23165231

[B16] Kang H. Hong Z. Zhong M. Klomp J. Bayless K. J. Mehta D. (2019). Piezo1 Mediates Angiogenesis through Activation of MT1-MMP Signaling. Am. J. Physiology-Cell Physiol. 316 (1), C92–c103. 10.1152/ajpcell.00346.2018 PMC6383143 30427721

[B17] Lamouille S. Xu J. Derynck R. (2014). Molecular Mechanisms of Epithelial-Mesenchymal Transition. Nat. Rev. Mol. Cell Biol 15 (3), 178–196. 10.1038/nrm3758 24556840 PMC4240281

[B18] Lee K. Nelson C. M. (2012). New Insights into the Regulation of Epithelial-Mesenchymal Transition and Tissue Fibrosis. Int. Rev. Cel. Mol. Biol. 294, 171–221. 10.1016/b978-0-12-394305-7.00004-5 22364874

[B19] Li C. Rezania S. Kammerer S. Sokolowski A. Devaney T. Gorischek A. (2015). Piezo1 Forms Mechanosensitive Ion Channels in the Human MCF-7 Breast Cancer Cell Line. Sci. Rep. 5, 8364. 10.1038/srep08364 25666479 PMC4322926

[B20] Li N. He Y. Yang G. Yu Q. Li M. (2019). Role of TRPC1 Channels in Pressure-Mediated Activation of Airway Remodeling. Respir. Res. 20 (1), 91. 10.1186/s12931-019-1050-x 31092255 PMC6518742

[B21] Liu J. Yang Y. Liu Y. (2021). Piezo1 Plays a Role in Optic Nerve Head Astrocyte Reactivity. Exp. Eye Res. 204, 108445. 10.1016/j.exer.2021.108445 33465396 PMC7946740

[B22] Liu L. Wu N. Wang Y. Zhang X. Xia B. Tang J. (2019). TRPM7 Promotes the Epithelial-Mesenchymal Transition in Ovarian Cancer through the Calcium-Related PI3K/AKT Oncogenic Signaling. J. Exp. Clin. Cancer Res. 38 (1), 106. 10.1186/s13046-019-1061-y 30819230 PMC6396458

[B23] Mai X. Shang J. Liang S. Yu B. Yuan J. Lin Y. (2016). Blockade of Orai1 Store-Operated Calcium Entry Protects against Renal Fibrosis. Jasn 27 (10), 3063–3078. 10.1681/asn.2015080889 26940090 PMC5042666

[B24] Mittal V. (2018). Epithelial Mesenchymal Transition in Tumor Metastasis. Annu. Rev. Pathol. Mech. Dis. 13, 395–412. 10.1146/annurev-pathol-020117-043854 29414248

[B25] Mousawi F. Peng H. Li J. Ponnambalam S. Roger S. Zhao H. (2020). Chemical Activation of the Piezo1 Channel Drives Mesenchymal Stem Cell Migration via Inducing ATP Release and Activation of P2 Receptor Purinergic Signaling. Stem cells (Dayton, Ohio) 38 (3), 410–421. 10.1002/stem.3114 PMC7064961 31746084

[B26] Nisticò P. Bissell M. J. Radisky D. C. (2012). Epithelial-mesenchymal Transition: General Principles and Pathological Relevance with Special Emphasis on the Role of Matrix Metalloproteinases. Cold Spring Harb Perspect. Biol. 4 (2). 10.1101/cshperspect.a011908 PMC3281569 22300978

[B27] Nourse J. L. Pathak M. M. (2017). How Cells Channel Their Stress: Interplay between Piezo1 and the Cytoskeleton. Semin. Cel. Dev. Biol. 71, 3–12. 10.1016/j.semcdb.2017.06.018 PMC6070642 28676421

[B28] Orlichenko L. S. Radisky D. C. (2008). Matrix Metalloproteinases Stimulate Epithelial-Mesenchymal Transition during Tumor Development. Clin. Exp. Metastasis 25 (6), 593–600. 10.1007/s10585-008-9143-9 18286378

[B29] Przybyla L. Muncie J. M. Weaver V. M. (2016). Mechanical Control of Epithelial-To-Mesenchymal Transitions in Development and Cancer. Annu. Rev. Cell Dev. Biol. 32, 527–554. 10.1146/annurev-cellbio-111315-125150 27298087

[B30] Qin Y. Capaldo C. Gumbiner B. M. Macara I. G. (2005). The Mammalian Scribble Polarity Protein Regulates Epithelial Cell Adhesion and Migration through E-Cadherin. J. Cel. Biol. 171 (6), 1061–1071. 10.1083/jcb.200506094 PMC2171311 16344308

[B31] Savagner P. (2001). Leaving the Neighborhood: Molecular Mechanisms Involved during Epithelial-Mesenchymal Transition. Bioessays 23 (10), 912–923. 10.1002/bies.1132 11598958

[B32] Segel M. Neumann B. Hill M. F. E. Weber I. P. Viscomi C. Zhao C. (2019). Niche Stiffness Underlies the Ageing of central Nervous System Progenitor Cells. Nature 573 (7772), 130–134. 10.1038/s41586-019-1484-9 31413369 PMC7025879

[B33] Sharma S. Goswami R. Zhang D. X. Rahaman S. O. (2019). TRPV 4 Regulates Matrix Stiffness and TGF β1‐induced Epithelial‐mesenchymal Transition. J. Cell Mol Med 23 (2), 761–774. 10.1111/jcmm.13972 30450767 PMC6349341

[B34] Sim J. Y. Moeller J. Hart K. C. Ramallo D. Vogel V. Dunn A. R. (2015). Spatial Distribution of Cell-Cell and Cell-ECM Adhesions Regulates Force Balance while Main-taining E-C-adherin M-olecular T-ension in C-ell P-airs. MBoC 26 (13), 2456–2465. 10.1091/mbc.e14-12-1618 25971797 PMC4571300

[B35] Solis A. G. Bielecki P. Steach H. R. Sharma L. Harman C. C. D. Yun S. (2019). Mechanosensation of Cyclical Force by PIEZO1 Is Essential for Innate Immunity. Nature 573 (7772), 69–74. 10.1038/s41586-019-1485-8 31435009 PMC6939392

[B36] Stewart T. A. Davis F. M. (2019). Formation and Function of Mammalian Epithelia: Roles for Mechanosensitive PIEZO1 Ion Channels. Front. Cell Dev. Biol. 7, 260. 10.3389/fcell.2019.00260 31750303 PMC6843007

[B37] Sugisawa E. Takayama Y. Takemura N. Kondo T. Hatakeyama S. Kumagai Y. (2020). RNA Sensing by Gut Piezo1 Is Essential for Systemic Serotonin Synthesis. Cell 182 (3), 609–624. e21. 10.1016/j.cell.2020.06.022 32640190

[B38] Thiery J. P. Acloque H. Huang R. Y. J. Nieto M. A. (2009). Epithelial-mesenchymal Transitions in Development and Disease. Cell 139 (5), 871–890. 10.1016/j.cell.2009.11.007 19945376

[B39] Uttamsingh S. Bao X. Nguyen K. T. Bhanot M. Gong J. Chan J. L.-K. (2008). Synergistic Effect between EGF and TGF-Β1 in Inducing Oncogenic Properties of Intestinal Epithelial Cells. Oncogene 27 (18), 2626–2634. 10.1038/sj.onc.1210915 17982486

[B40] Wang Y. Xiao B. (2018). The Mechanosensitive Piezo1 Channel: Structural Features and Molecular Bases Underlying its Ion Permeation and Mechanotransduction. J. Physiol. 596 (6), 969–978. 10.1113/jp274404 29171028 PMC5851880

[B41] Xu J. Lamouille S. Derynck R. (2009). TGF-β-induced Epithelial to Mesenchymal Transition. Cell Res 19 (2), 156–172. 10.1038/cr.2009.5 19153598 PMC4720263

[B42] Yang Z. Zhang X. Gang H. Li X. Li Z. Wang T. (2007). Up-regulation of Gastric Cancer Cell Invasion by Twist Is Accompanied by N-Cadherin and Fibronectin Expression. Biochem. biophysical Res. Commun. 358 (3), 925–930. 10.1016/j.bbrc.2007.05.023 17512904

[B43] Yilmaz M. Christofori G. (2009). EMT, the Cytoskeleton, and Cancer Cell Invasion. Cancer Metastasis Rev. 28 (1-2), 15–33. 10.1007/s10555-008-9169-0 19169796

[B44] Yu-Ju Wu C. Chen C.-H. Lin C.-Y. Feng L.-Y. Lin Y.-C. Wei K.-C. (2020). CCL5 of Glioma-Associated Microglia/macrophages Regulates Glioma Migration and Invasion via Calcium-dependent Matrix Metalloproteinase 2. Neuro-oncology 22 (2), 253–266. 10.1093/neuonc/noz189 31593589 PMC7032635

[B45] Zeisberg M. Neilson E. G. (2009). Biomarkers for Epithelial-Mesenchymal Transitions. J. Clin. Invest. 119 (6), 1429–1437. 10.1172/jci36183 19487819 PMC2689132

[B46] Zhou J. Wang J. Zhang N. Zhang Y. Li Q. (2015). Identification of Biomechanical Force as a Novel Inducer of Epithelial-Mesenchymal Transition Features in Mechanical Stretched Skin. Am. J. Transl Res. 7 (11), 2187–2198. 26807167 PMC4697699

[B47] Zhou Y. Li H. Liang X. Du H. Suo Y. Chen H. (2020). The CCN1 (CYR61) Protein Promotes Skin Growth by Enhancing Epithelial‐mesenchymal Transition during Skin Expansion. J. Cell Mol Med 24 (2), 1460–1473. 10.1111/jcmm.14828 31828970 PMC6991652

